# Female infertility from oocyte maturation arrest: assembling the genetic puzzle

**DOI:** 10.15252/emmm.202317729

**Published:** 2023-04-19

**Authors:** Xinbao Ding, John C Schimenti

**Affiliations:** ^1^ Department of Biomedical Sciences, College of Veterinary Medicine Cornell University Ithaca NY USA

**Keywords:** Genetics, Gene Therapy & Genetic Disease, Urogenital System

## Abstract

Assisted reproduction procedures often encounter an issue called oocyte maturation arrest (OMA), which is manifested as failed IVF/ICSI attempts using oocytes from some infertile women. In this issue of *EMBO Molecular Medicine*, Wang *et al* identify infertile women bearing novel DNA sequence variants in a gene called *PABPC1L*, which is essential for translation of maternal mRNAs. By conducting a series of *in vitro* and *in vivo* experiments, they demonstrated certain variants as being causal for OMA, confirming a conserved requirement for PABPC1L in human oocyte maturation. This study offers a promising therapeutic target for treating OMA patients.

## Graphical abstract

J. Schimenti and X. Ding discuss the study by Weijie Wang *et al* (in this issue of *EMBO Mol Med*) that reports novel pathogenic variants in PABPC1L that cause oocyte maturation arrest and female infertility.

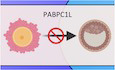

Infertility is a heterogeneous condition, and the genetic factors account for about half of all cases. Next‐generation sequencing, including whole genome and exome sequencing (WGS and WES), has significantly hastened the identification of candidate disease‐causing variants (population polymorphisms) or private mutations. Validation of candidates is essential for accurate diagnosis and potential clinical treatment. Whereas nonsense or frameshift variants have a high likelihood of being deleterious, interpreting missense variants is much more challenging. These can affect protein characteristics in several ways, including tertiary structure, stability, activity, macromolecule interactions, and post‐translational modifications. *In silico* predictors used for classifying missense variants often produce false pathogenicity calls for alleles including those in infertility genes (preprint: Ding *et al*, 2021). Therefore, it is critical to experimentally validate predictions using biochemical or functional assays in cultured cells or genome‐edited animal models. The latter is particularly important for potential infertility alleles impacting gamete development or function because it is not yet feasible to accurately recapitulate gametogenesis *in vitro*. In this issue of *EMBO Molecular Medicine*, Wang *et al* (2023) present a comprehensive pipeline to identify and interpret pathogenic variants from infertility patients, using a degree of rigor that is exceptional for the reproductive genetics field.

In their study, Wang *et al* (2023) aimed to identify the genetic causes of OMA, a disease that results in female infertility and is a major contributor to *in vitro* fertilization failure. OMA can manifest at different stages of meiosis, and a few genes functioning in diverse processes such as recombination, spindle assembly, zona pellucida formation, and translational repression have been linked to OMA in women (Huang *et al*, 2014; Chen *et al*, 2017; Liu *et al*, 2017; Maddirevula *et al*, 2017; Dai *et al*, 2019; Zhang *et al*, 2020). Given the large number of genes that cause meiotic arrest in mice when ablated but not yet definitely linked to human infertility, it is likely that variants/mutations in scores of genes can cause OMA. The authors focused on *PABPC1L* (poly(A)‐binding protein cytoplasmic 1‐like) encoding a protein that binds to the elongated poly(A) tail to stabilize polyadenylated mRNAs. Female mice deficient in *Pabpc1l* are infertile; oocytes are dysmorphic and do not complete maturation due to impaired translational activation of maternal mRNAs (Guzeloglu‐Kayisli *et al*, 2012). In the ClinVar database, a pathogenic missense variant in *PABPC1L* was reported in 3 sisters from a consanguineous Turkish family with OMA; however, no functional evidence was provided, underscoring a common shortcoming in the implication of human infertility variants.

Wang *et al* (2023) conducted a thorough evaluation of potential OAM variants in *PABPC1L*. The study followed a four‐step approach (Fig 1). Firstly, they recruited and characterized individuals with OMA. Secondly, they performed WES on the patients and their families to discover and prioritize high‐confidence variants based on bioinformatic prediction of molecular consequence, inheritance pattern in families, *in silico* prediction, and allele frequency. Their prioritized candidates included one nonsense variant, four missense variants, one frameshift mutant, and a 3′UTR variant. Thirdly, the authors conducted *in vitro* experiments in HeLa cells to test whether any of the variants impacted protein behavior. They found that the truncating variants (nonsense and frameshift alleles) altered protein stability and localization, and the 3′UTR variant affected expression of a reporter. Whereas none of missense variants exhibited such defects, three of the four tested caused a dramatic decrease in the binding to, and translation of, the mRNA of a luciferase reporter. Fourthly, they constructed knock‐in (KI) mouse lines harboring 3 of the human orthologous missense variants using CRISPR‐Cas9 genome editing. All the KI female mice were infertile and exhibited early embryo arrest prior to the blastocyst stages, including those bearing the allele (p.Arg374Gln) that did not confer a significant *in vitro* defect. This demonstrates the importance *in vivo* modeling, lest pathogenic variants are dismissed as benign or vice versa.

**Figure 1:**
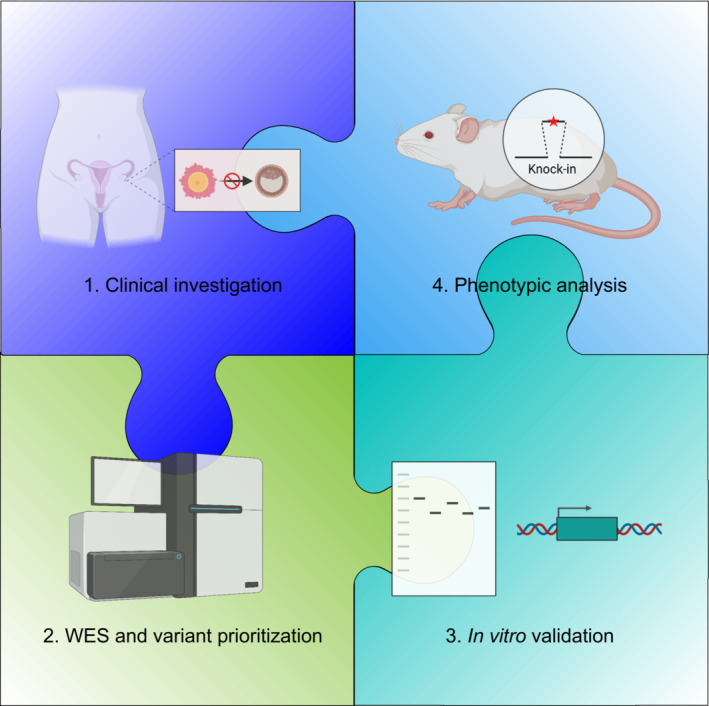
Unpuzzling PABPC1L variants in women with oocyte maturation arrest This study utilized a systematic process to confirm the genetic causality of PABPC1L variants in patients with OMA. It was accomplished by assembling four pieces of puzzle: (1) clinical investigations of patient history and IVF phenotype; (2) sequencing and *in silico* analysis to identify candidate variants; (3) *in vitro* analysis of PABPC1L variants for altered behavior; and (4) generation and analysis of mouse models for variants. The figure was created in part using BioRender.

Having proven causality of the variants, the authors performed transcriptome analysis of KI mouse zygotes to elucidate the mechanisms underlying maturation arrest in PABPC1L‐deficient oocytes. This revealed upregulation of the Mos‐MAPK pathway. Activation of this pathway is important for inducing MII arrest of oocytes, and the authors went on to show that microinjection of WT human *MOS* cRNA (complementary RNA) into WT mouse zygotes phenocopied the KI phenotype.

The confirmation of genetic causality, coupled with the studies implicating MOS overexpression in PABPC1L variant oocytes, paves the way for potential therapeutic approaches. The oocytes or early embryos from a woman bearing pathogenic *PABPC1L* variants might be “rescued” by introducing Mos siRNA or MAPK inhibitors. An alternative approach could model previous studies in mice where microinjection of *Pabpc1l* mRNA into preantral follicle‐enclosed *Pabpc1l* ^−/−^ oocytes rescued oocyte maturation (Lowther & Mehlmann, 2015), although this approach did not work with denuded GV oocytes (Guzeloglu‐Kayisli *et al*, 2012).

Overall, this paper stands out as being exemplary in the field of human reproductive genetics in that it rigorously and convincingly solved the puzzle of infertility for several women harboring candidate variants in their genome, unlike most variant reports that stop at step 2 as illustrated in Fig 1, thereby leaving key pieces of the puzzle missing (steps 3 and 4). Even more impressive is that the authors used mechanistic and functional studies to offer a potential pathway for treatment for patients with these *PABPC1L* variants. Not all genes will be as amenable to the sorts of analyses that so neatly solved the puzzle here, but the bar has been set.
